# Inflammatory Prognostic Markers in COPD: Clinical Utility of CAR and MGPS

**DOI:** 10.3390/jcm15166372

**Published:** 2026-08-18

**Authors:** Mustafa Düger, Güzide Tomas, Şeyma Başlılar

**Affiliations:** 1 Department of Pulmonology, Istanbul Mega Medipol Hospital, Istanbul Medipol University, Istanbul 34214, Türkiye; 2 Department of Pulmonology, Sultan Abdulhamid Han Training and Research Hospital, Health Sciences University, Istanbul 34668, Türkiye; guzide.tomas@hotmail.com (G.T.); seymabaslilar@yahoo.com (Ş.B.)

**Keywords:** COPD, CAR, modified Glasgow Prognostic Score (mGPS), prognostic biomarkers, systemic inflammation

## Abstract

**Background**: Acute exacerbations of chronic obstructive pulmonary disease (AECOPD) are associated with substantial morbidity and mortality, highlighting the need for simple and reliable biomarkers for early risk stratification. The C-reactive protein to albumin ratio (CAR) and the modified Glasgow Prognostic Score (mGPS) reflect systemic inflammation and nutritional status, but their prognostic value in hospitalized patients with AECOPD remains incompletely defined. This study aimed to evaluate the associations of CAR and mGPS with one-year mortality and indicators of disease severity in hospitalized patients with AECOPD. **Methods**: In this retrospective single-center cohort study, 1556 adult patients hospitalized with a primary diagnosis of AECOPD between January 2020 and January 2026 were included. Patients with concomitant pneumonia and other major inflammatory conditions were excluded. Demographic, clinical, laboratory, arterial blood gas, and pulmonary function data were retrospectively analyzed. CAR was calculated from admission C-reactive protein and serum albumin levels, whereas mGPS was determined according to established criteria. Independent predictors of one-year mortality were identified using multivariable logistic regression analyses. Receiver operating characteristic (ROC) curve analysis was performed to evaluate the discriminatory performance of CAR. **Results**: During the one-year follow up, 140 patients (9.0%) died. Compared with survivors, non-survivors had significantly higher CRP levels, higher CAR values, lower serum albumin levels, more severe hypercapnia and acidosis, and higher rates of intensive care unit admission and invasive mechanical ventilation (all *p* < 0.001). The distribution of mGPS differed significantly according to mortality status, with patients in the mGPS 2 category exhibiting the highest mortality rates (*p* < 0.001). After adjustment for clinically relevant covariates, age, acidosis, invasive mechanical ventilation, and CAR remained independently associated with one-year mortality. CAR demonstrated excellent discriminatory performance for predicting one-year mortality (AUC 0.907, 95% CI 0.893–0.922; *p* < 0.001), with an optimal cut-off value of >3.96, yielding 98.6% sensitivity and 84.3% specificity. Increasing mGPS scores were associated with progressively worse clinical outcomes, including higher rates of intensive care unit admission, invasive mechanical ventilation, in-hospital mortality, and one-year mortality. **Conclusions**: Inflammation and nutrition-based biomarkers are closely associated with disease severity and one-year mortality in hospitalized patients with AECOPD. CAR remained an independent predictor of one-year mortality and demonstrated excellent discriminatory performance, supporting its role as a simple and readily available complementary biomarker for prognostic assessment. Increasing mGPS scores were associated with progressively worse clinical outcomes, suggesting that mGPS may also contribute to risk stratification in hospitalized patients with AECOPD. Prospective multicenter studies with external validation are warranted to confirm these findings and to further define the prognostic value of these biomarkers.

## 1. Introduction

COPD is a heterogeneous and progressive respiratory disorder characterized by persistent respiratory symptoms, airflow limitation, recurrent exacerbations, and substantial morbidity and mortality. According to recent Global Initiative for Chronic Obstructive Lung Disease (GOLD) reports and global epidemiological data, COPD remains one of the leading causes of death worldwide and represents a major public health burden due to frequent hospitalizations, exacerbations, reduced quality of life, and increased healthcare utilization [1,2]. The clinical course of COPD is highly variable among individuals, and identifying patients at high risk for adverse outcomes remains an important challenge in daily clinical practice.

In recent years, systemic inflammation has been increasingly recognized as a key component of COPD pathophysiology and disease progression. Beyond airway inflammation, elevated circulating inflammatory biomarkers have been associated with exacerbation frequency, impaired lung function, increased comorbidity burden, prolonged hospitalization, and mortality [3,4]. Growing evidence suggests that circulating inflammation-related biomarkers have prognostic value across a wide spectrum of chronic inflammatory diseases, reflecting disease activity and systemic inflammatory burden beyond organ-specific manifestations [5]. Among these biomarkers, CRP, an acute phase reactant synthesized in response to systemic inflammation, has been extensively investigated in COPD and has shown prognostic relevance in both stable disease and acute exacerbations [6].

However, inflammation alone may not fully reflect the complex clinical status of patients with COPD. Nutritional impairment and cachexia are also important determinants of disease severity and prognosis. Serum albumin, a routinely measured laboratory parameter, reflects nutritional status, systemic inflammation, and physiological reserve. Hypoalbuminemia has been associated with poor outcomes in chronic respiratory diseases and critically ill patients [7]. Therefore, combining inflammatory and nutritional markers may provide a more comprehensive assessment of disease burden and prognosis.

The CAR has recently emerged as a simple and inexpensive composite biomarker reflecting both systemic inflammation and nutritional status. Previous studies have demonstrated that elevated CAR levels are associated with worse prognosis in several chronic inflammatory and malignant diseases [8,9]. In COPD, Shen et al. reported that higher CAR levels were independently associated with increased mortality risk, suggesting that CAR may be a useful marker for risk stratification in COPD patients [10]. Furthermore, recent population-based studies have supported the association between elevated CAR and the presence and severity of COPD [11].

Another inflammation-based prognostic marker, the mGPS, combines CRP and albumin levels into a simple scoring system reflecting systemic inflammatory response and nutritional decline. mGPS has been widely evaluated in oncology and chronic inflammatory conditions and has consistently demonstrated prognostic significance [11]. Nevertheless, data regarding the prognostic value of mGPS in COPD remain limited and inconsistent. Some studies suggest that inflammation-based prognostic indices may predict mortality and intensive care requirement in severe COPD, whereas others report weaker associations depending on patient population and disease severity [12].

Although both CAR and mGPS have previously been investigated in patients with COPD, several important knowledge gaps remain. Most published studies have evaluated these biomarkers separately, have focused on either stable COPD or selected critically ill populations, or have included relatively small cohorts with limited adjustment for clinically relevant confounders. Furthermore, evidence evaluating the prognostic performance of both CAR and mGPS within the same cohort of hospitalized patients with AECOPD remains limited. Therefore, the relative prognostic utility of these two inexpensive inflammation and nutrition-based biomarkers for predicting clinically relevant outcomes, including one-year mortality, has not been fully established. Addressing this gap may help clarify their complementary roles in risk stratification and facilitate the identification of patients at increased risk for adverse outcomes.

Hospitalized patients with COPD, particularly those requiring ICU admission or ventilatory support, represent a clinically vulnerable population with significantly increased short- and long-term mortality risk. Early identification of high-risk patients using inexpensive and easily accessible biomarkers could contribute to more effective clinical monitoring, individualized treatment strategies, and optimized healthcare resource utilization.

Therefore, in this retrospective cohort study, we aimed not only to evaluate the prognostic significance of CAR and mGPS in hospitalized patients with AECOPD but also to compare their clinical utility within the same large real-world cohort. Specifically, we investigated their associations with one-year mortality, intensive care unit requirement, mechanical ventilation, blood gas abnormalities, and other indicators of disease severity. We hypothesized that these readily available inflammation and nutrition-based biomarkers would provide complementary prognostic information for risk stratification in hospitalized patients with AECOPD.

## 2. Materials and Methods

### 2.1. Study Design and Population

This retrospective, single-center cohort study was conducted at the Department of Pulmonology and the Respiratory Intensive Care Unit (RICU) of Sultan II Abdülhamid Han Training and Research Hospital, Türkiye. Adult patients hospitalized with a primary diagnosis of acute exacerbation of AECOPD between 1 January 2020 and 1 January 2026 were retrospectively screened for eligibility.

The diagnosis of AECOPD was established according to the GOLD criteria and was defined as an acute worsening of respiratory symptoms requiring additional medical treatment and hospitalization after exclusion of alternative causes of acute respiratory deterioration.

Only hospitalized patients were included in the study. Patients admitted either directly to the pulmonology ward or to the RICU were eligible. Patients initially admitted to the pulmonology ward who subsequently required transfer to the RICU because of clinical deterioration were also included. Outpatients and emergency department patients who were discharged without hospitalization were not included.

For patients with multiple hospitalizations during the study period, only the first hospitalization fulfilling the inclusion criteria was considered the index admission and included in the analyses to avoid duplication and within-patient correlation.

### 2.2. Inclusion and Exclusion Criteria

Patients were eligible for inclusion if they met all of the following criteria:

- Aged 18 years or older;
- Hospitalized with a primary diagnosis of acute exacerbation of AECOPD between 1 January 2020 and 1 January 2026;
- A diagnosis of COPD confirmed by previous spirometry according to the Global Initiative for Chronic Obstructive Lung Disease (GOLD) criteria;
- Had available serum CRP and albumin measurements obtained at hospital admission before initiation of treatment;
- Had available pulmonary function test (PFT) data performed during the stable phase of COPD before the index hospitalization;
- Complete clinical records and completed at least one year of follow up after the index hospitalization.

Patients were excluded if they had the following:

- Radiologically confirmed pneumonia at admission;
- Active pulmonary tuberculosis or any other active bacterial, viral, or fungal infection;
- Hematological disorders or hematological malignancies;
- Chronic liver disease or liver failure;
- Active autoimmune or systemic inflammatory diseases;
- Parenteral nutritional support before hospitalization;
- Immunosuppressive therapy, including systemic corticosteroid treatment for diseases other than COPD;
- Or incomplete or unverifiable clinical records.
- Patients with radiographic evidence of pneumonia on chest radiography or thoracic computed tomography at admission were excluded.

### 2.3. Data Collection

Clinical, laboratory, and follow up data were retrospectively obtained from the hospital electronic medical record system and archived patient files. Only patients who had completed at least one year of follow up after the index hospitalization were eligible for inclusion in the study, ensuring complete ascertainment of the predefined one-year mortality outcome for all included patients. Mortality status was verified using both hospital records and the national death registry whenever available. For patients with multiple hospitalizations during the study period, only data from the index hospitalization were included in the analyses. Although patients had longer available follow up within the electronic health record system, the primary endpoint was predefined as all-cause mortality occurring within one year of the index hospitalization.

### 2.4. Demographic and Clinical Variables

The following demographic and clinical characteristics were recorded:

- Age;
- Sex;
- Smoking status and cumulative smoking exposure (pack years);
- Body mass index (BMI);
- Comorbid diseases;
- Hospitalization location (pulmonology ward or respiratory intensive care unit);
- Length of hospital stay;
- Requirement for non-invasive mechanical ventilation (NIMV);
- Requirement for invasive mechanical ventilation (IMV);
- In-hospital mortality;
- One-year all-cause mortality;
- Duration of follow up;
- Number of COPD exacerbations during follow up.

### 2.5. Laboratory Parameters

All laboratory parameters were obtained from blood samples collected at hospital admission before initiation of medical treatment for the index AECOPD episode.

The following laboratory variables were recorded:

- C-reactive protein (CRP);
- Serum albumin;
- Hemoglobin;
- Total leukocyte count;
- Platelet count;
- Neutrophil count;
- Lymphocyte count;
- Eosinophil count.

The CAR was calculated by dividing the serum CRP concentration by the serum albumin concentration.

The modified Glasgow Prognostic Score (mGPS) was calculated according to previously established criteria:

- **mGPS = 0:** CRP ≤ 1.0 mg/dL
- **mGPS = 1:** CRP > 1.0 mg/dL and albumin ≥3.5 g/dL (≥35 g/L)
- **mGPS = 2:** CRP > 1.0 mg/dL and albumin <3.5 g/Dl (<35 g/L)

#### Pulmonary Function Tests and Blood Gas Parameters

Pulmonary function test parameters, including forced expiratory volume in one second (FEV_1_), forced vital capacity (FVC), FEV_1_/FVC ratio, predicted FEV_1_ (%), and predicted FVC (%), were obtained from the most recent spirometry performed during the stable phase of COPD before the index hospitalization.

Arterial blood gas measurements, including pH, partial pressure of carbon dioxide (PaCO_2_), partial pressure of oxygen (PaO_2_), and arterial oxygen saturation (SaO_2_), were obtained at hospital admission before initiation of treatment. For categorical analyses, acidosis was defined as arterial pH < 7.35, hypercapnia as PaCO_2_ > 45 mmHg, and hypoxemia as PaO_2_ < 60 mmHg, according to standard clinical definitions.

### 2.6. Study Outcomes

The primary outcome of the study was all-cause mortality within one year following the index hospitalization for AECOPD. Follow up was initiated on the date of the index hospital admission and continued for one year. Deaths occurring during the index hospitalization were included in the primary outcome.

Secondary outcomes included RICU admission, the requirement for NIMV, the requirement for IMV, arterial blood gas abnormalities, and pulmonary function impairment as indicators of disease severity.

### 2.7. Statistical Analysis

Statistical analyses were performed using IBM SPSS Statistics for MacOS version 30.0 (IBM Corp., Armonk, NY, USA). The distribution of continuous variables was assessed using the Kolmogorov–Smirnov test. In addition, skewness and kurtosis values within ±2 were considered supportive of the assumption of normality. Categorical variables were presented as number and percentage [*n* (%)], whereas continuous variables were expressed as mean ± standard deviation (SD) or median (interquartile range [IQR]), according to their distribution.

For comparisons between two groups, Student’s *t*-test was used for normally distributed continuous variables, whereas Welch’s *t*-test was applied when the assumption of homogeneity of variances was not met. The Mann–Whitney U test was used for non-normally distributed continuous variables. For comparisons among three groups, one-way analysis of variance (ANOVA) was used for normally distributed variables, with Welch ANOVA applied when variance homogeneity was violated. Non-normally distributed variables were compared using the Kruskal–Wallis test. When overall group differences were statistically significant, post hoc analyses were performed using the Bonferroni, Games–Howell, or Dunn–Bonferroni tests, as appropriate. Categorical variables were compared using the Pearson chi-square test or Fisher’s exact test, when appropriate.

Univariate and multivariable logistic regression analyses were performed to identify independent predictors of 1-year mortality. Variables that were statistically significant in the univariate analysis and those considered clinically relevant were entered into the multivariable models. To minimize potential multicollinearity, highly correlated variables were not included in the same model. Accordingly, CRP, serum albumin, and the CRP/albumin ratio were not entered simultaneously into the same multivariable model. Similarly, only one of the variables acidosis, pH, and PaCO_2_ was included in each model. Results were reported as odds ratios (ORs) with 95% confidence intervals (CIs).

Because the mGPS demonstrated complete separation for mortality, it could not be reliably evaluated using conventional logistic regression and was therefore excluded from the multivariable analysis. Likewise, pH was excluded because its inclusion as a continuous variable caused model instability.

Patients with missing data for variables included in the analyses were excluded from the corresponding analyses (complete case analysis), and no data imputation procedures were performed.

The discriminatory performance of the CRP/albumin ratio for predicting 1-year mortality was evaluated using receiver operating characteristic (ROC) curve analysis. The area under the curve (AUC) and its 95% confidence interval were calculated. The optimal cut-off value was determined using the Youden index, and the corresponding sensitivity and specificity were reported.

All statistical tests were two-tailed, and a *p*-value < 0.05 was considered statistically significant.

### 2.8. Ethical Approval

The study protocol was approved by the Non Interventional Clinical Research Ethics Committee of Sultan II. Abdülhamid Han Training and Research Hospital. Due to the retrospective design of the study, informed consent was waived. All procedures were conducted in accordance with the principles of the Declaration of Helsinki.

## 3. Results

A total of 1556 hospitalized patients with acute exacerbation of COPD were included in the study. During the 1-year follow up, 1416 patients (91.0%) survived, whereas 140 patients (9.0%) died. The demographic, clinical, laboratory, arterial blood gas, and pulmonary function characteristics of the patients according to 1-year mortality status are presented in Table 1.

Patients who died within one year were significantly older than survivors (74 ± 11 vs. 72 ± 12 years, *p* = 0.014) and had a higher BMI (27.5 ± 5.4 vs. 26.4 ± 5.3 kg/m^2^, *p* = 0.012). No significant differences were observed between survivors and non-survivors regarding sex, smoking status, smoking exposure, comorbidities, chronic heart disease, chronic kidney disease, diabetes mellitus, hypoxemia, biomass exposure, or ward length of stay.

Regarding clinical characteristics, acidosis, hypercapnia, ICU admission, NIMV requirement, and IMV requirement were all significantly more frequent among non-survivors (all *p* < 0.001). Follow-up duration was also significantly longer in patients who died within one year (*p* < 0.001), whereas ICU length of stay did not differ significantly between the groups.

Laboratory analyses demonstrated that serum CRP levels and the CRP/albumin ratio (CAR) were significantly higher, whereas serum albumin levels were significantly lower among non-survivors (all *p* < 0.001). Among hematological parameters, eosinophil percentage, eosinophil count, and lymphocyte count were significantly lower in the mortality group, while platelet, neutrophil, and monocyte counts did not differ significantly between the groups.

Arterial blood gas analyses showed significantly higher PaCO_2_ levels and lower pH values among patients who died within one year (both *p* < 0.001). Although PaO_2_ values also differed significantly between groups (*p* = 0.015), oxygen saturation was comparable. Pulmonary function parameters, including FEV_1_, FVC, FEV_1_/FVC ratio, predicted FEV_1_, and predicted FVC, were not significantly associated with 1-year mortality.

The distribution of mGPS categories differed significantly according to 1-year mortality status (*p* < 0.001). Patients with an mGPS score of 2 accounted for the highest proportion of deaths (82.9% of non-survivors), whereas no deaths occurred among patients with an mGPS score of 0.

The results of the univariate and multivariable logistic regression analyses are presented in Table 2. In the univariate analysis, older age, higher BMI, acidosis, hypercapnia, NIMV requirement, IMV requirement, lower serum albumin levels, higher CRP levels, increased CAR, higher PaCO_2_ levels, and lower eosinophil counts were significantly associated with 1-year mortality.

In multivariable Model 1, age, acidosis, IMV requirement, and CAR remained independently associated with 1-year mortality. Each one unit increase in CAR was associated with a 16% increase in the odds of 1-year mortality (OR = 1.16, 95% CI: 1.07–1.26, *p* < 0.001). IMV requirement was the strongest independent predictor of mortality (OR = 44.30, 95% CI: 21.91–89.61, *p* < 0.001).

Similarly, in Model 2, age, IMV requirement, and CAR remained independently associated with mortality, whereas BMI and PaCO_2_ were no longer statistically significant.

The demographic, clinical, laboratory, arterial blood gas, and pulmonary function characteristics according to mGPS categories are summarized in Table 3. Patients with higher mGPS scores were significantly older (*p* < 0.001) and showed higher frequencies of acidosis, ICU admission, NIMV requirement, IMV requirement, in-hospital mortality, and 1-year mortality (all *p* ≤ 0.003). Ward length of stay also increased significantly across mGPS categories (*p* = 0.001).

Regarding laboratory findings, increasing mGPS scores were associated with progressively higher CRP levels, higher CAR values, higher neutrophil and monocyte counts, and lower albumin, lymphocyte, eosinophil count, eosinophil percentage, and Prognostic Nutritional Index (PNI) (all *p* ≤ 0.010). Smoking status, PaCO_2_, and pH also differed significantly among mGPS groups, whereas sex, BMI, smoking exposure, comorbidities, chronic heart disease, chronic kidney disease, diabetes mellitus, hypercapnia, hypoxemia, biomass exposure, ICU length of stay, follow-up duration, platelet count, oxygen saturation, and pulmonary function parameters did not differ significantly.

**Figure 1:**
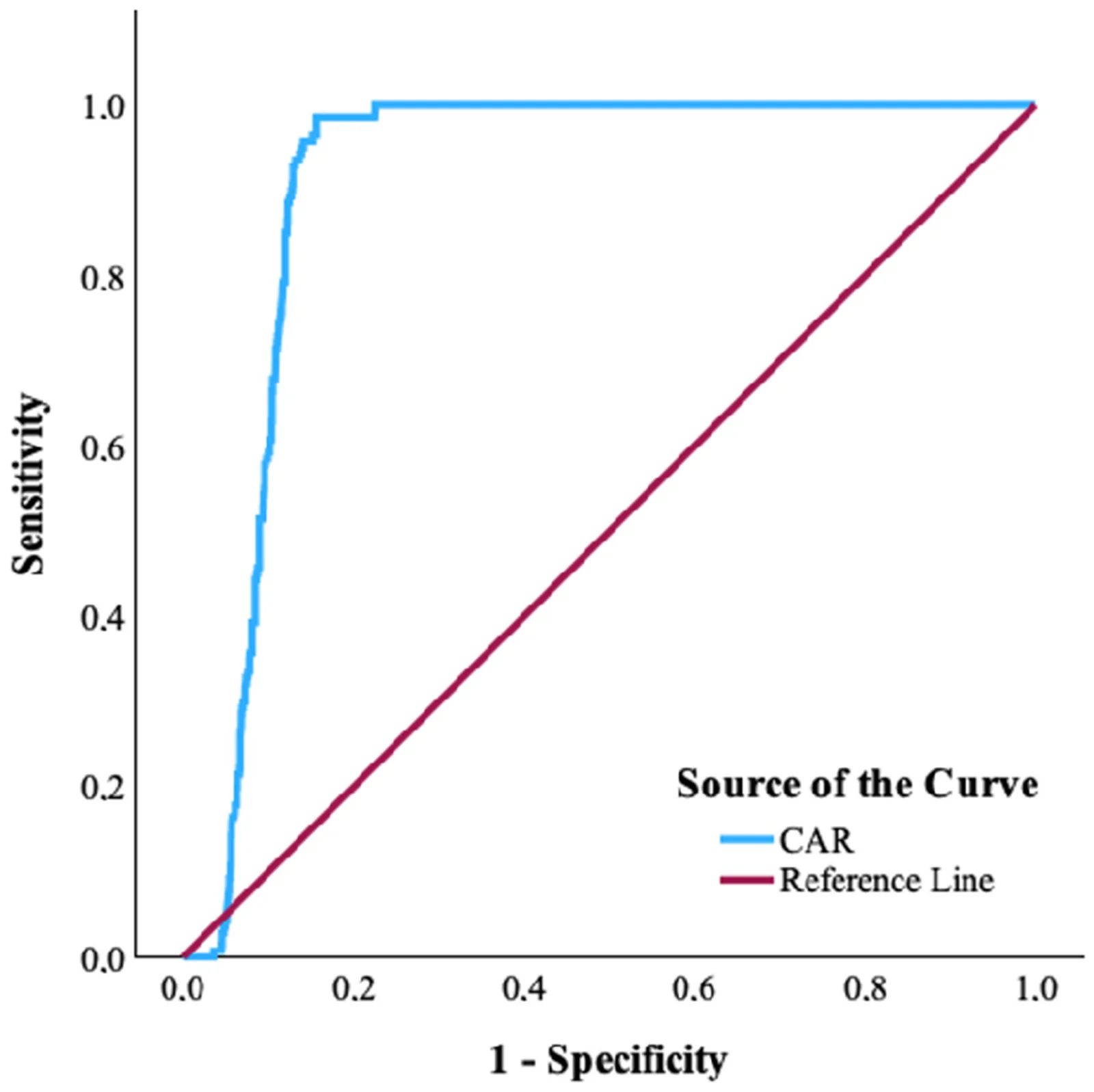
ROC Curve of the CRP/Albumin Ratio for Predicting 1-Year Mortality.

The ROC curve analysis evaluating the discriminatory performance of CAR for predicting 1-year mortality is presented in Table 4 and Figure 1. CAR demonstrated excellent discriminative ability, with an AUC of 0.907 (95% CI: 0.893–0.922, *p* < 0.001). The optimal cut-off value was >3.96, providing a sensitivity of 98.6% and a specificity of 84.3% for predicting 1-year mortality.

**Model 1** included age, BMI, acidosis, IMV requirement, and the CRP/albumin ratio.

**Model 2** included age, BMI, IMV requirement, PaCO_2_, and the CRP/albumin ratio.

The **mGPS** variable was not included in the multivariate analysis because it showed complete separation for mortality, whereas **pH** was excluded because it caused model instability.

Results are presented as **odds ratios (ORs) with 95% confidence intervals (CIs)**.

## 4. Discussion

In this retrospective cohort of patients hospitalized with acute exacerbations of COPD without concomitant pneumonia, we demonstrated that inflammation and nutrition-based biomarkers derived from routinely available laboratory parameters were closely associated with disease severity and one-year mortality. Patients who died within one year had significantly higher CRP levels, lower serum albumin levels, higher CAR values, and more severe respiratory failure, reflected by hypercapnia, acidosis, and an increased need for intensive care and invasive mechanical ventilation. After adjustment for clinically relevant covariates, CAR remained an independent predictor of one-year mortality. In addition, increasing mGPS scores were associated with progressively worse clinical outcomes, including higher rates of intensive care unit admission, invasive mechanical ventilation, in-hospital mortality, and one-year mortality. Overall, these findings suggest that inflammation and nutrition-based biomarkers may complement conventional clinical assessment for prognostic evaluation in hospitalized patients with acute exacerbations of COPD.

COPD is increasingly recognized as a chronic systemic inflammatory disorder rather than a disease confined solely to the airways. Persistent systemic inflammation contributes to skeletal muscle dysfunction, malnutrition, oxidative stress, endothelial dysfunction, and progressive clinical deterioration. Consequently, inflammation-based biomarkers have attracted growing interest as practical tools for risk stratification in patients with COPD. A recent study by Ao et al. further demonstrated a significant association between the CAR and the presence and severity of COPD, supporting the concept that composite inflammatory biomarkers may better reflect disease burden than single laboratory parameters alone [13]. Consistent with these observations, our findings suggest that systemic inflammatory burden is closely associated with adverse clinical outcomes, particularly among patients hospitalized with acute exacerbations of COPD. The parallel increase in CAR and deterioration in mGPS across patient groups further supports the close interplay between systemic inflammation, nutritional impairment, and adverse clinical outcomes in AECOPD.

Among the evaluated biomarkers, CAR demonstrated particularly strong prognostic significance. The biological rationale for CAR is compelling because it simultaneously reflects two critical mechanisms involved in COPD progression: systemic inflammation and impaired nutritional reserve. CRP is an acute-phase reactant that increases in response to inflammatory cytokines, whereas serum albumin decreases during chronic inflammation, oxidative stress, and catabolic states. Consequently, CAR may provide a more comprehensive reflection of overall disease burden than either parameter alone. Our findings are consistent with those reported by Shen and Xiao, who demonstrated that elevated CAR was independently associated with increased mortality in patients with COPD [8]. Similarly, Guan and Weng reported that higher CAR values were associated with greater disease severity and poorer prognosis in hospitalized patients with COPD [14]. Collectively, these findings support the potential utility of CAR as an inexpensive, readily available biomarker for identifying patients at increased risk of adverse outcomes.

A notable finding of our study was the excellent predictive ability of CAR for one-year mortality, with a cut-off value of >3.96 showing very high sensitivity and specificity. These findings suggest that CAR may serve as a practical adjunctive biomarker for risk stratification in hospitalized patients with acute exacerbation of COPD. Given that both CRP and albumin are routinely available laboratory parameters, CAR represents a simple, inexpensive, and reproducible index that may complement established clinical assessment rather than replace existing prognostic tools. Early identification of high-risk patients using CAR may facilitate closer monitoring, timely therapeutic interventions, and appropriate decisions regarding the need for intensive care support. Although the optimal role of CAR within existing COPD risk assessment strategies requires further investigation, our findings suggest that a CAR value >3.96 may serve as an adjunctive indicator to identify patients who may benefit from closer clinical monitoring, more intensive supportive care, and early recognition of disease progression. However, CAR should not be considered a substitute for established clinical assessment or validated prognostic tools but rather a complementary biomarker that may improve overall risk stratification. Future prospective studies directly comparing CAR with established COPD prognostic models are warranted to determine its incremental prognostic value.

We also found that increasing mGPS scores were associated with progressively worse clinical outcomes in hospitalized patients with AECOPD. Patients with higher mGPS scores had significantly higher rates of ICU admission, invasive mechanical ventilation, in-hospital mortality, and 1-year mortality. In addition, increasing mGPS scores were accompanied by higher CRP levels, higher CRP/albumin ratios, lower serum albumin concentrations, lower Prognostic Nutritional Index (PNI) values, and less favorable hematological profiles, reflecting a greater systemic inflammatory burden and poorer nutritional status.

The mGPS has been widely investigated as a prognostic marker in patients with malignancies and chronic inflammatory disorders, whereas evidence in COPD remains relatively limited. Because mGPS integrates both systemic inflammation and nutritional status into a simple and inexpensive scoring system, it may provide clinically relevant prognostic information beyond individual laboratory parameters. Our findings support this concept by demonstrating a stepwise deterioration in clinical outcomes with increasing mGPS scores, suggesting that mGPS may be useful for identifying hospitalized patients with AECOPD who are at increased risk of adverse outcomes. Similar observations have been reported in previous studies evaluating inflammation- and albumin-based prognostic indices in COPD, although their independent prognostic performance has varied across different study populations [15]. Taken together, these findings suggest that mGPS may serve as a practical complementary biomarker for risk stratification in hospitalized patients with AECOPD. Nevertheless, prospective multicenter studies are warranted to further validate its prognostic value and to determine its incremental utility alongside established clinical risk assessment tools.

The observed associations between hypercapnia, acidosis, and mortality are supported by well-established mechanisms regulating ventilatory control. Under physiological conditions, increases in PaCO_2_ and decreases in arterial pH stimulate central chemoreceptors within the medulla, leading to an increase in ventilatory drive. Peripheral chemoreceptors located in the carotid bodies also respond to hypoxemia and contribute to compensatory increases in ventilation. However, in advanced COPD, chronic CO_2_ retention may attenuate central chemoreceptor sensitivity, resulting in greater dependence on peripheral hypoxic stimulation. During acute exacerbations, progressive hypercapnia and respiratory acidosis reflect failure of these compensatory mechanisms, respiratory muscle fatigue, and impaired gas exchange, ultimately increasing the likelihood of ventilatory failure and the need for invasive mechanical ventilation. These physiological mechanisms provide a plausible explanation for the strong associations between hypercapnia, acidosis, respiratory failure, and mortality observed in our cohort [16,17].

Another important observation was the strong association between respiratory failure parameters and mortality. Hypercapnia, acidosis, and particularly the requirement for invasive mechanical ventilation were significantly associated with poor outcomes. In the multivariable analysis, invasive mechanical ventilation emerged as the strongest independent predictor of 1-year mortality. These findings are biologically plausible because hypercapnia reflects advanced respiratory failure and has been recognized as an independent predictor of adverse outcomes in COPD. In a comprehensive review, Csoma et al. emphasized that chronic hypercapnia contributes not only to impaired gas exchange but also to epithelial dysfunction, immune dysregulation, cardiovascular complications, and skeletal muscle wasting, all of which may contribute to increased mortality. Consistent with these observations, hypercapnia was significantly associated with mortality in our univariate analysis, while the need for invasive mechanical ventilation remained the strongest independent predictor after multivariable adjustment [18]. This finding is clinically plausible, as the need for invasive ventilatory support generally reflects severe respiratory failure, advanced physiological deterioration, and a high burden of acute illness during COPD exacerbations. Similar findings have been reported in previous studies of patients hospitalized with severe AECOPD, in which the severity of respiratory failure and the requirement for ventilatory support consistently predicted both short- and long-term mortality [19]. These findings emphasize that inflammation-based biomarkers such as CAR and mGPS should be interpreted as complementary to, rather than replacements for, established clinical indicators of disease severity. From a clinical perspective, elevated CAR, impaired physiological reserve, hypercapnic respiratory failure, and the requirement for invasive mechanical ventilation may represent complementary features of a high-risk clinical phenotype in hospitalized patients with AECOPD. However, because the interactions among these variables were not directly evaluated in the present study, this interpretation should be considered hypothesis-generating rather than conclusive.

Interestingly, spirometric parameters, including FEV_1_ and FVC, were not significantly associated with one-year mortality in our cohort. Although airflow limitation remains fundamental for the diagnosis and classification of COPD, increasing evidence suggests that spirometric impairment alone does not fully capture the systemic inflammatory burden or the prognosis of patients hospitalized with acute exacerbations. Furthermore, the pulmonary function test results analyzed in the present study represented the most recent stable-state measurements obtained before hospitalization rather than measurements performed during the acute exacerbation, which may partly explain their limited prognostic value in this setting. Recent multidimensional prognostic studies have emphasized the importance of integrating inflammatory, nutritional, hematologic, and functional biomarkers into comprehensive risk assessment models for COPD patients [20]. Our findings are consistent with this evolving concept and suggest that inflammation-based biomarkers may complement conventional physiological assessment in hospitalized patients with AECOPD.

The present study has several strengths. It included a large real-world cohort of patients hospitalized specifically for acute exacerbations of COPD, with comprehensive clinical, laboratory, arterial blood gas, and pulmonary function data. Importantly, patients with concomitant pneumonia and other major inflammatory conditions were excluded, thereby reducing potential confounding effects on CRP and albumin-based biomarkers. In addition, detailed comorbidity data were incorporated into the analyses, allowing a more comprehensive evaluation of the independent prognostic performance of CAR and mGPS. Finally, the evaluated biomarkers are inexpensive, rapidly obtainable, and routinely available in daily clinical practice, supporting their potential applicability as complementary tools for prognostic assessment.

## 5. Limitations

Several limitations of this study should be acknowledged. First, the retrospective, single-center design may have introduced selection bias and may limit the generalizability of the findings. Second, inflammatory biomarkers were evaluated only at the time of hospital admission; therefore, dynamic changes in CAR and mGPS during hospitalization and follow-up could not be assessed. Third, although significant associations between inflammation-based biomarkers and mortality were identified, causal relationships cannot be established because of the observational design. Furthermore, mortality data were obtained from the Turkish Ministry of Health national electronic health record system (e-Nabız) as a binary one-year outcome (alive/dead), whereas exact dates of death were not consistently available. Therefore, time-to-event analyses, such as Cox proportional hazards regression and Kaplan–Meier survival analysis, could not be performed. Fourth, formal model calibration and internal validation procedures, such as bootstrapping or cross validation, were not performed. Therefore, although the multivariable model demonstrated excellent discriminative performance, the possibility of model overfitting cannot be completely excluded, and the predictive performance of the model should be confirmed in independent external cohorts. Fifth, although patients with concomitant pneumonia and other major inflammatory conditions were excluded to minimize confounding, our findings may not be generalizable to all patients hospitalized with COPD exacerbations. Finally, despite adjustment for important clinical variables and comorbidities, residual confounding from unmeasured factors, including COPD severity (e.g., standardized GOLD stage), long-term maintenance pharmacotherapy, prior exacerbation history, nutritional interventions, medication adherence, and exacerbation phenotypes, cannot be completely excluded. Because this retrospective study covered an approximately ten-year period during which COPD classification and treatment recommendations evolved, standardized retrospective determination of GOLD stage for all patients was not considered sufficiently reliable and therefore was not included in the analyses.

## 6. Conclusions

In conclusion, this study demonstrated that inflammation- and nutrition-based biomarkers derived from routinely available laboratory parameters are closely associated with disease severity and one-year mortality in patients hospitalized with acute exacerbations of COPD without concomitant pneumonia. Among the evaluated biomarkers, the CRP-to-albumin ratio (CAR) remained an independent predictor of one-year mortality and showed excellent discriminative performance, supporting its potential value as a complementary tool for prognostic assessment. In addition, increasing mGPS scores were associated with progressively worse clinical outcomes, including higher rates of intensive care unit admission, invasive mechanical ventilation, in-hospital mortality, and one-year mortality. Given their simplicity, low cost, and widespread availability, CAR and mGPS may complement established clinical assessment in identifying patients at increased risk of poor outcomes. Nevertheless, prospective multicenter studies incorporating external validation are warranted to confirm these findings and to further define the incremental prognostic value of CAR and mGPS alongside established clinical risk assessment tools in patients with acute exacerbations of COPD.

## Figures and Tables

**Table 1:** Comparison of Demographic, Clinical, and Laboratory Characteristics According to 1-Year Mortality Status.

Variables	1-Year Mortality			*p*-Value
	Total (N = 1556)	Survived (*n* = 1416)	Non-survivors (*n* = 140)	
Sex, *n* (%)				0.269
Male	1012 (65)	915 (64.6)	97 (69.3)	
Female	544 (35)	501 (35.4)	43 (30.7)	
**Age (years)**	**72 ± 12**	**72 ± 12**	**74 ± 11**	**0.014**
**BMI (kg/m^2^)**	**26.5 ± 5.3**	**26.4 ± 5.3**	**27.5 ± 5.4**	**0.012**
**Acidosis, *n* (%)**	**445 (28.6)**	**334 (23.6)**	**111 (79.3)**	**<0.001**
**Hypercapnia, *n* (%)**	**1042 (67)**	**918 (64.8)**	**124 (88.6)**	**<0.001**
Hypoxemia, *n* (%)	841 (54)	768 (54.2)	73 (52.1)	0.635
Ward length of stay (days)	6 (3.9–9.0)	6 (3.9–8.9)	6.2 (4.0–9.9)	0.290
**ICU requirement, *n* (%)**	**242 (15.6)**	**102 (7.2)**	**140 (100)**	**<0.001**
**NIMV requirement, *n* (%)**	**537 (34.5)**	**436 (30.8)**	**101 (72.1)**	**<0.001**
**IMV requirement, *n* (%)**	**114 (7.3)**	**21 (1.5)**	**93 (66.4)**	**<0.001**
ICU length of stay (days)	10.7 ± 6.6	10.5 ± 6.6	11.9 ± 6.7	0.423
**Follow up duration (years)**	**3.5 ± 1.0**	**3.5 ± 1.0**	**4.1 ± 0.7**	**<0.001**
**In hospital mortality, *n* (%)**	**52 (3.3)**	**52 (3.7)**	**0 (0)**	**0.012**
**Modified Glasgow Prognostic Score (mGPS), *n* (%)**				**<0.001**
0	374 (24.0)	374 (26.4)	0 (0.0)	
1	442 (28.4)	418 (29.5)	24 (17.1)	
2	740 (47.6)	624 (44.1)	116 (82.9)	
**Eosinophil percentage (%)**	**0.5 (0.1–1.8)**	**0.5 (0.1–1.9)**	**0.3 (0–1.0)**	**0.008**
Neutrophil count (×10^3^/µL)	7.1 (5.4–9.8)	7.1 (5.4–9.8)	7.2 (5.5–9.6)	0.927
**Lymphocyte count (×10^3^/µL)**	**1.2 (0.7–1.8)**	**1.2 (0.7–1.8)**	**1.1 (0.7–1.6)**	**0.018**
Monocyte count (×10^3^/µL)	0.6 (0.4–0.9)	0.6 (0.4–0.9)	0.5 (0.3–0.9)	0.349
**Eosinophil count (×10^3^/µL)**	**0.1 (0–0.2)**	**0.1 (0–0.2)**	**0 (0–0.1)**	**0.003**
**Albumin (g/L)**	**32.4 ± 6.8**	**32.9 ± 6.7**	**27.9 ± 5.9**	**<0.001**
**C-reactive protein (CRP) (mg/L)**	**39.5 (10.7–112.9)**	**32.1 (8.9–84.0)**	**158 (135–183)**	**<0.001**
**CRP/Albumin ratio**	**1.2 (0.3–3.6)**	**1.0 (0.3–2.7)**	**5.8 (5.1–6.7)**	**<0.001**
**PaCO_2_ (mmHg)**	**51.3 ± 12.8**	**50.0 ± 11.4**	**65.4 ± 17.6**	**<0.001**
**pH**	**7.4 ± 0.1**	**7.4 ± 0.1**	**7.3 ± 0.1**	**<0.001**
**PaO_2_ (mmHg)**	**59 (48–79.2)**	**58.8 (48–78.8)**	**61.6 (51.2–87.1)**	**0.015**
Oxygen saturation (%)	90.2 (82–96)	90.2 (82–96)	90.2 (82.8–96)	0.842
Prognostic Nutritional Index (PNI)	35.3 (30.3–39.6)	35.2 (30.2–39.6)	36.1 (30.7–39.4)	0.628

Abbreviations: BMI, body mass index; ICU, intensive care unit; NIMV, non-invasive mechanical ventilation; IMV, invasive mechanical ventilation; mGPS, modified Glasgow Prognostic Score; CRP, C-reactive protein; PNI, prognostic nutritional index; PaCO_2_, partial pressure of carbon dioxide; PaO_2_, partial pressure of oxygen. Note: Categorical variables are presented as *n* (*%*), normally distributed continuous variables as mean ± standard deviation (SD), and non-normally distributed continuous variables as median (interquartile range [IQR]).

**Table 2:** Univariate and Multivariate Logistic Regression Analyses of Factors Associated with 1-Year Mortality.

Variables	Univariate Analysis		Multivariate Analysis (Model 1)		Multivariate Analysis (Model 2)	
	OR (95% CI)	*p*-Value	OR (95% CI)	*p*-Value	OR (95% CI)	*p*-Value
**Age (years)**	**1.02 (1.00–1.04)**	**0.014**	**1.03 (1.01–1.06)**	**0.006**	**1.04 (1.01–1.06)**	**0.004**
BMI (kg/m^2^)	1.04 (1.01–1.07)	0.012	1.02 (0.98–1.07)	0.359	1.02 (0.97–1.07)	0.416
Acidosis	12.40 (8.09–19.00)	<0.001	2.45 (1.37–4.37)	0.002		
Hypercapnia	4.20 (2.47–7.16)	<0.001				
NIMV requirement	5.82 (3.96–8.57)	<0.001				
**IMV requirement**	**131.44 (75.41–229.11)**	**<0.001**	**44.30 (21.91–89.61)**	**<0.001**	**69.19 (29.83–160.50)**	**<0.001**
**Albumin (g/L)**	**0.90 (0.87–0.92)**	**<0.001**				
**C-reactive protein (CRP) (mg/L)**	**1.014 (1.012–1.017)**	**<0.001**				
**CRP/Albumin ratio**	**1.42 (1.35–1.51)**	**<0.001**	**1.16 (1.07–1.26)**	**<0.001**	**1.17 (1.08–1.27)**	**<0.001**
**PaCO_2_ (mmHg)**	**1.09 (1.07–1.10)**	**<0.001**			1.00 (0.98–1.03)	0.724
Lymphocyte count (×10^3^/µL)	1.04 (0.97–1.11)	0.239				
**Eosinophil count (×10^3^/µL)**	**0.26 (0.08–0.81)**	**0.020**				

Abbreviations: OR, odds ratio; CI, confidence interval; BMI, body mass index; NIMV, non-invasive mechanical ventilation; CRP, C-reactive protein; PaCO_2_, partial pressure of carbon dioxide. Note: In the univariate analysis, each variable was evaluated separately. Variables considered clinically relevant and significantly associated with mortality in the univariate analysis were included in the multivariate models.

**Table 3:** Comparison of Demographic, Clinical, and Laboratory Characteristics According to mGPS.

Variables	mGPS-0 (*n* = 374)	mGPS-1 (*n* = 442)	mGPS-2 (*n* = 740)	*p*-Value
Sex, *n* (%)				0.918
Male	244 (65.2)	284 (64.3)	484 (65.4)	
Female	130 (34.8)	158 (35.7)	256 (34.6)	
**Age (years)**	**70 ± 12**	**71 ± 11**	**73 ± 11**	**<0.001**
BMI (kg/m^2^)	26.2 ± 5.1	26.6 ± 5.4	26.5 ± 5.3	0.473
**Acidosis, *n* (%)**	**82 (21.9)**	**128 (29.0)**	**235 (31.8)**	**0.003**
Hypercapnia, *n* (%)	243 (65.0)	297 (67.2)	502 (67.8)	0.626
Hypoxemia, *n* (%)	219 (58.6)	225 (50.9)	397 (53.6)	0.088
Known biomass exposure, *n* (%)	39 (10.4)	55 (12.4)	97 (13.1)	0.433
**Ward length of stay (days)**	**5.4 (3.7–8.0)**	**5.8 (3.9–8.2)**	**6.3 (4.0–9.8)**	**0.001**
**ICU requirement, *n* (%)**	**0 (0)**	**29 (6.6)**	**213 (28.8)**	**<0.001**
**NIMV requirement, *n* (%)**	**114 (30.5)**	**132 (29.9)**	**291 (39.3)**	**<0.001**
**IMV requirement, *n* (%)**	**0 (0)**	**21 (4.8)**	**93 (12.6)**	**<0.001**
ICU length of stay (days)	10.3 ± 5.9	11.2 ± 7.5	10.6 ± 6.5	0.862
Follow-up duration (years)	3.5 ± 1.0	3.6 ± 1.0	3.5 ± 0.9	0.255
**In-hospital mortality, *n* (%)**	**0 (0)**	**2 (0.5)**	**50 (6.8)**	**<0.001**
**1-year mortality, *n* (%)**	**0 (0)**	**24 (5.4)**	**116 (15.7)**	**<0.001**
Platelet count (×10^3^/µL)	240 (177–282)	240 (193–296)	236.5 (181.5–303)	0.415
**Eosinophil percentage (%)**	**0.9 (0.1–2.5)**	**0.4 (0.1–1.9)**	**0.5 (0.1–1.5)**	**0.003**
**Neutrophil count (×10^3^/µL)**	**6.2 (4.9–8.4)**	**7.1 (5.5–10.0)**	**7.7 (5.7–10.3)**	**<0.001**
**Lymphocyte count (×10^3^/µL)**	**1.4 (0.9–2.1)**	**1.3 (0.8–1.8)**	**1.1 (0.7–1.7)**	**<0.001**
**Monocyte count (×10^3^/µL)**	**0.5 (0.3–0.7)**	**0.6 (0.4–0.9)**	**0.6 (0.4–1.0)**	**<0.001**
**Eosinophil count (×10^3^/µL)**	**0.1 (0–0.2)**	**0 (0–0.2)**	**0 (0–0.2)**	**0.010**
**Albumin (g/L)**	**34.4 ± 7.1**	**38.7 ± 2.7**	**27.7 ± 4.5**	**<0.001**
**C-reactive protein (CRP) (mg/L)**	**4 (2–6.8)**	**43.9 (23.3–88.8)**	**85 (35.4–150.5)**	**<0.001**
**CRP/Albumin ratio**	**0.1 (0.1–0.2)**	**1.2 (0.6–2.3)**	**3.0 (1.2–5.8)**	**<0.001**
**PaCO_2_ (mmHg)**	**50.6 ± 10.5**	**50.4 ± 11.8**	**52.3 ± 14.4**	**0.016**
**pH**	**7.39 ± 0.05**	**7.38 ± 0.06**	**7.37 ± 0.08**	**0.001**
**PaO_2_ (mmHg)**	**56 (47–74.7)**	**60.5 (50–78.6)**	**59.2 (48–80)**	**0.002**
Oxygen saturation (%)	89.2 (82–95.4)	90.7 (81.7–96)	90.4 (82.6–96.4)	0.290
**Prognostic Nutritional Index (PNI)**	**37.1 (31.8–41.2)**	**39.7 (37.8–42.0)**	**31.2 (27.5–34.1)**	**<0.001**

Abbreviations: BMI, body mass index; ICU, intensive care unit; NIMV, non-invasive mechanical ventilation; IMV, invasive mechanical ventilation; mGPS, modified Glasgow Prognostic Score; CRP, C-reactive protein; PNI, prognostic nutritional index; PaCO_2_, partial pressure of carbon dioxide; PaO_2_, partial pressure of oxygen. Note: Categorical variables are presented as *n* (*%*), normally distributed continuous variables as *mean ± standard deviation (SD)*, and non-normally distributed continuous variables as *median (interquartile range [IQR]).*

**Table 4:** ROC Analysis of the CRP/Albumin Ratio for Predicting 1-Year Mortality.

Variable	AUC (95% CI)	*p*-Value	Cut-Off Value	Sensitivity (%)	Specificity (%)
CRP/Albumin ratio	0.907 (0.893–0.922)	<0.001	>3.96	98.6	84.3

Abbreviations: AUC, area under the curve; CI, confidence interval; CRP, C-reactive protein. Note: The optimal cut-off value was determined using the Youden index.

## Data Availability

The datasets used and/or analyzed during the current study are available from the corresponding author on reasonable request. The data are not publicly available due to ethical restrictions and the need to protect patients’ privacy.

## Abbreviations

COPD	Chronic Obstructive Pulmonary Disease
CAR	C-reactive Protein to Albumin Ratio
mGPS	Modified Glasgow Prognostic Score
CRP	C-reactive Protein
ICU	Intensive Care Unit
NIMV	Non-Invasive Mechanical Ventilation
MV	Mechanical Ventilation
RICU	Respiratory Intensive Care Unit
PFT	Pulmonary Function Test
FEV1	Forced Expiratory Volume in 1 Second
FVC	Forced Vital Capacity
ROC	Receiver Operating Characteristic
AUC	Area Under the Curve
OR	Odds Ratio
CI	Confidence Interval
